# Novel technology at hand to measure skin hydration by Biodisplay smartphone touch screen panel

**DOI:** 10.1038/s41598-021-98784-1

**Published:** 2021-09-30

**Authors:** YoungHwan Choi, Se Jin Oh, Jong Hee Lee

**Affiliations:** 1 Department of Dermatology, Samsung Medical Center, Sungkyunkwan University School of Medicine, 81 Irwon-ro, Gangnam-gu, Seoul, 06351 Republic of Korea; 2 Department of Medical Device Management and Research, SAIHST, Sungkyunkwan University, Seoul, Korea

**Keywords:** Biotechnology, Health care

## Abstract

Skin hydration is generally evaluated using devices that measure capacitance or conductance. A new technology (Biodisplay) was developed to provide accurate measurements of skin hydration at the contact site. This study aimed to test the reliability of the Biodisplay by comparing its performance results with those of similar devices currently used to objectively assess skin hydration. For each of the 30 participants, skin hydration was measured at each of the defined points on the forearm three times using the Biodisplay and a Hydration probe (HP), an objective measurement device of skin hydration. We also evaluated skin hydration of the arm using both tools after applying moisturizers to evaluate interferences from skin care products. The reliability and reproducibility of each device were analyzed by intraclass correlation coefficients (ICC), and the correlation of the two devices was evaluated by Pearson’s correlation coefficients (PCC). The Biodisplay demonstrated moderate-to-excellent reliability (ICC: (0.741–0.980)), but lower reliability than the HP (ICC: (0.953–0.980)). The skin hydration measurements made by the two devices were demonstrated to be significantly correlated, showing moderate correlations (PCC: 0.601). The Biodisplay can provide reasonably reliable and accurate measurements for skin hydration with the strong points of portability and accessibility.

## Introduction

Personalized medicine is becoming more important. Since the smartphone has the advantage of high accessibility, smartphone-based devices and applications can play an important role in personalized medicine. The use of the smartphone as a healthcare device has attracted interest in recent years. The main target is a smartphone-based method that enables blood pressure and blood sugar level control, as well as checking of the heart rate and other variables that are essential for maintaining health. Skin is another excellent target for smartphone applications. The smartphone can become a daily skin care device in the era of personalized medicine. As the smartphone market has continued growing, various mobile applications to evaluate and manage skin health have been developed^1^. In 2012, it was reported that there were more than 200 skin management applications available^2^. Since the skin is the outermost organ of the human body, it is easy to evaluate or measure its characteristics using camera-based devices, such as smartphones. Recently, as people have become increasingly interested in their skin health, devices to measure the skin have also been actively developed, such as camera-based devices. Smartphone-based applications have been developed to measure wrinkles, pigmentation, and erythema, and their usefulness has been validated^3^. Although skin hydration can reflect the skin barrier function, which is one of the most important factors in skin health and management, it is difficult to measure skin hydration using smartphones themselves or camera-based devices.

Certain conventional methods are used to measure skin hydration. For example, with an instrument emitting a high frequency of 3.5 MHz, the skin hydration state can be evaluated using either skin conductance (the reciprocal of resistance) or capacitance. The Corneometer (Courage + Khasaka, Cologne, Germany) and the DermaLab Combo(Cortex Technology, Hadsund, Denmark) measure skin capacitance or conductance based on water content, and are widely used devices to measure skin hydration in an objective manner^4–6^.

Many bio-sensing applications have been reported, and these have used various smartphone modules, such as cameras, light emitting diodes, and infrared sensors, but there has been no application using touchscreen panels. The touch screen panel in a smartphone recognizes the coordinates of touch by sensing the variance of capacitance, and this has been dominantly utilized for the user interface or user experience purposes. Biodisplay, a novel concept for measuring skin hydration, uses a new approach that reflects the skin moisture condition by simply using the touch screen panel of a smartphone. It is innovative from the perspective that it measures skin hydration by using only the touch screen panel, without the need for any additional attached or connected devices or sensors. The mechanical and electrical properties of the skin are modulated by changes in skin hydration^7^. The current technology allows capacitance to be quantitatively measured at the contact site, through which a bio-algorithm can be developed that converts the capacitance data into skin hydration data. Since skin hydration is one of the primary research interests in the fields of cosmetics and dermatology, this technology may lead to new possibilities for measuring skin hydration using a smartphone at hand.

This study was designed to evaluate the efficacy of the Biodisplay as an objective and reliable skin moisture measuring device, Hydration probe (HP).

## Results

In total, 30 participants were included in this study (Table 1). Of these, 13 (43.3%) were male, and 17 (56.7%) were female. The mean age was 38.2 (range (24–52) years).

**Table 1:** Demographics of the participants

Characteristics	
Total participants (no.)	30
**Sex**	
Male (no.)	13
Female (no.)	17
Male : female	1:1.31
Age (years)	38.2

*No* number of patients, *y* year

### Skin hydration measurement using Biodisplay and HP

This study was designed to evaluate whether the measurement of skin hydration using Biodisplay is useful in everyday life, we measured skin hydration for 3 conditions: before moisturizer application, after moisturizer application and after removing remnant moisturizer. In each condition, three measurements were done to evaluate the reproducibility of each device and the reliability of Biodisplay by comparing the data of Biodisplay and HP.

We calculated the average of the skin hydration values measured by each device (Table 2). The skin hydration values of the arm measured by Biodisplay and HP ranged from 21.64 to 85.15 (mean 58.07, standard deviation 11.29) and from 97.67 to 462.67 μS (mean 229.01, standard deviation 68.21), respectively. Before applying moisturizer, the skin hydration values of the arm measured by Biodisplay and HP ranged from 19.77 to 73.85 (mean 51.69, standard deviation 12.56) and from 95 to 406 μS (mean 207.90, standard deviation 73.75), respectively. After applying moisturizer, the skin hydration values of the arm measured by Biodisplay and HP ranged from 38.53 to 91.16 (mean 59.91, standard deviation 9.99) and from 166 to 477 μS (mean 225.84, standard deviation 65.03) respectively. After removing the moisturizer, the skin hydration values of the arm measured by Biodisplay and HP ranged from 29.10 to 89.54 (mean 62.61, standard deviation 10.68) and from 181 to 444 μS (mean 253.29, standard deviation 60.79), respectively.

**Table 2:** Average skin hydration values of Biodisplay and hydration probe.

	Device	Skin hydration value
Overall	Biodisplay	58.07
	Hydration probe (μS)	229.01
Before moisturizer	Biodisplay	51.69
	Hydration probe (μS)	207.90
After moisturizer	Biodisplay	59.91
	Hydration probe (μS)	225.84
After removing moisturizer	Biodisplay	62.61
	Hydration probe (μS)	253.29

### Test–retest reliability

#### Test–retest reliability of Biodisplay

The intraclass correlation coefficeints (ICC) determining the test–retest reliability of the skin hydration measurement of Biodisplay showed moderate to excellent agreement which ranged from 0.741 to 0.913 (Table 3). Excellent agreement was observed only in the arm before moisturizer (ICC = 0.953). Good agreement was observed in the arm overall (ICC = 0.897).

**Table 3:** Test–retest reliabilities of the Biodisplay and hydration probe.

	Device	Intraclass correlation coefficients
Overall	Biodisplay	0.897
	Hydration probe	0.969
Before moisturizer	Biodisplay	0.913
	Hydration probe	0.953
After moisturizer	Biodisplay	0.741
	Hydration probe	0.980
After removing moisturizer	Biodisplay	0.837
	Hydration probe	0.969

#### Test–retest reliability of the HP

The ICC determining the test–retest reliability of the skin hydration measurement of HP showed excellent agreement in all parts ranging from 0.953 to 0.980 (Table 3). The order of ICC was as follows, in descending order from the nearest to 1: arm after moisturizer (ICC = 0.980), arm after removing moisturizer (ICC = 0.969), arm overall (ICC = 0.969), and arm before moisturizer (ICC = 0.953).

#### Correlation between Biodisplay and the HP

**Figure 1:**
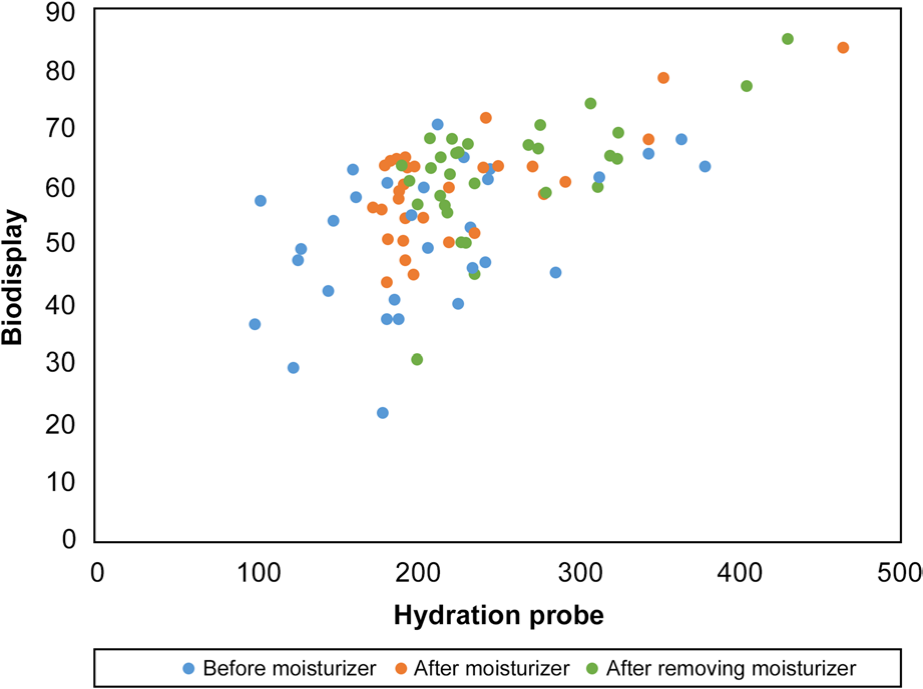
Scatter plot showing the relationships between the Biodisplay and hydration probe. Moderate correlation was observed between the two modalities (PCC = 0.601, *p* < 0.001).

The scatter plot and Pearson’s correlation coefficients (PCC) showed correlations between Biodisplay and HP (Fig. 1). The PCC results demonstrated that there was a significant correlation in the arm overall (*p *value < 0.001). A moderate correlation was observed, and the order of PCC was as follows, in descending order from the nearest to 1: after moisturizer application (PCC = 0.687), overall (PCC = 0.601), after removing moisturizer (PCC = 0.598) and before moisturizer (PCC = 0.474).

## Discussion

This study investigated the reproducibility of skin hydration measurements conducted and the correlation obtained with Biodisplay. The reproducibility of repetitive measurements can enhance the credibility of the application, which is particularly important for a smartphone application that is expected to be used every day. The skin hydration measurements obtained using Biodisplay were demonstrated to have a moderate to excellent test–retest reliability. When compared to Biodisplay, HP demonstrated better reliability for skin hydration measurement; this was expected, considering that HP is a standard method for evaluating the skin hydration status. In terms of intraclass correlation coefficients, Biodisplay produced better reproducible hydration data in the baseline status, which means that putting on moisturizers, as well as other cosmetics and makeup, can interfere with the exact measurement of skin hydration. To assess this effect, skin hydration was measured after the application of moisturizer in two conditions. The measurement was conducted 30 min after the application of moisturizer, as well as after wiping off any remnant moisturizer. Although the reproducibility of Biodisplay was lower in the case of the measurement without removing remnant moisturizer, the intraclass correlation coefficient was 0.741, which is not that low. After the application of moisturizer, the skin hydration level checked by Biodisplay increased from 51.69 to 62.61 on average, suggesting that Biodisplay can be a good modality for the evaluation of skin hydration.

Regarding the relationship between the two devices, statistically significant correlations were demonstrated between Biodisplay and HP. The overall correlation between the two devices was 0.601 (*p* < 0.001), showing moderate correlation. While Biodisplay calculates the capacitance of the whole contact area of the skin, HP cannot cover the whole area. Considering this discrepancy, the moderate correlation between the two devices can be considered to be reasonable. Therefore, Biodisplay can be used to monitor skin hydration with reasonable reliability and accuracy.

Recent studies have introduced a number of new devices that can measure skin hydration^8–11^. Grinich et al. reported on a novel device for measuring stratum corneum hydration that showed precise and reliable measurements, compared to the currently used devices^10^. Since the device has sensors for measuring skin capacitance and can transmit data to a smartphone application, it can analyze skin barrier function at low cost^10^. Logger et al. described a novel device for measuring skin hydration that can also be used for clinical purposes^11^. They measured skin hydration in rosacea patients before and after treatment, and found that skin hydration in rosacea patients was impaired before treatment, but restored after treatment^11^. They suggested that a home-based skin barrier monitoring device could be used in the future for inflammatory skin diseases, specifically to improve care and compliance^11^. The major difference between Biodisplay and other new devices is that Biodisplay simply uses the display panel of the smartphone itself, without the need for the additional sensors to measure skin hydration. Therefore, Biodisplay can be more convenient and accessible than other home-based skin hydration measuring devices.

There are many advantages to a skin measuring device that uses a smartphone. For example, Kim et al. reported that smartphone-based imaging systems could provide high portability, and enable continuous quantitative monitoring^12^. They suggested that smartphone-based devices have the potential to serve as valuable healthcare tools for the early diagnosis of dermatologic diseases, as well as prognosis monitoring at home after treatment^12^. Further, according to Manahan et al., skin self-examination using mobile teledermoscopy is promising for the surveillance of lesions, as it provides accurate telediagnosis, and could aid screening and surveillance efforts^13^. All previous smartphone-based devices and applications were based on a camera system. To date, there have been no devices for measuring skin hydration based on the smartphone itself.

Skin hydration level is important both for everyday skin care, and for making selections among different cosmetics. Skin hydration level can also reflect skin barrier function. It is disrupted in many dermatologic diseases, including atopic dermatitis and psoriasis; the degree of disruption may correlate with disease activity. Patients who can measure their skin hydration using an easily accessible device can take more interest in their skin condition, and manage their skin health more actively. In this study, skin hydration measurements by Biodisplay showed reliability, without the need for additional devices or sensors. Since Biodisplay can measure skin hydration through a smartphone display panel, we suggest that Biodisplay is highly advantageous in terms of portability and accessibility. Therefore, it is expected that Biodisplay can be used for daily skin care in healthy individuals, and that it can also improve the adherence of patients with dermatologic disease by providing them with information about their skin barrier function. Furthermore, skin hydration information measured by Biodisplay can be utilized by other healthcare platforms for personalized medicine or health care.

Facial skin is likely the most measured area of skin hydration in the field of cosmetic dermatology and daily skin care. Biodisplay uses a smartphone display panel, and when it is used for facial evaluation, the contact skin area can vary due to facial curvature. Therefore, we designed this study using the volar forearm first. Previous studies have indicated that the volar forearm is representative of the face for measuring skin hydration and biomechanical properties, and that it is relevant for the assessment of the efficacy of cosmetic products ultimately destined for facial use^14,15^. This study shows the usefulness of Biodisplay for measuring skin hydration. Future studies should extend the application of Biodisplay to facial evaluation.

In this study, we evaluated Biodisplay for measuring skin hydration. Biodisplay showed reasonably reliable and accurate measurements. Since Biodisplay can measure skin hydration using only a smartphone display panel, Biodisplay is highly advantageous in terms of portability and accessibility. With these properties, we suggest that Biodisplay has an innovative technology that can be used in daily skin care and personalized medicine. We hope that this study will expand the usage of smartphone-based devices in dermatologic fields as well.

## Methods

This study was a prospective clinical trial that was approved by the institutional review board of Samsung Medical Center, Sungkyunkwan University School of Medicine in Seoul, Korea (2020-04-028). This study has been registered in Clinical Research Information Service on June 18, 2020 (http://cris.nih.go.kr, KCT0005146). The study was conducted according to the protocols of the Declaration of Helsinki.

### Study populations

The participants in this study were enrolled based on the following inclusion and exclusion criteria: Subjects who were between the ages of 20 and 60 years, and who were familiar with using smartphone applications. Subjects were excluded if they had a diagnosis of eczema or hyperhidrosis, or a history of any disease requiring treatment at the evaluated sites. All the included patients signed written informed consent for their participation.

### Devices

#### Biodisplay

**Figure 2:**
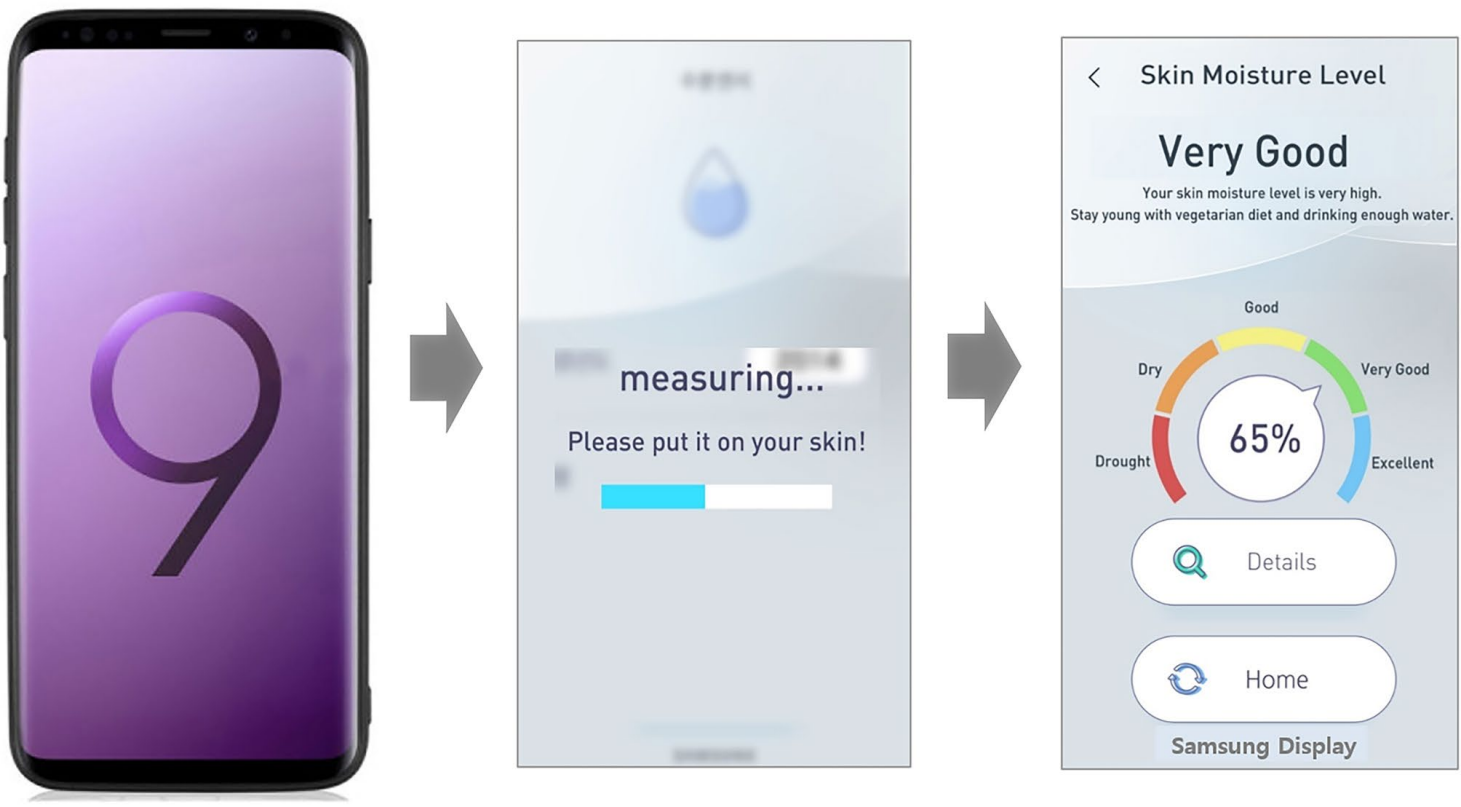
Scheme for skin hydration measurement using smartphone.

**Figure 3:**
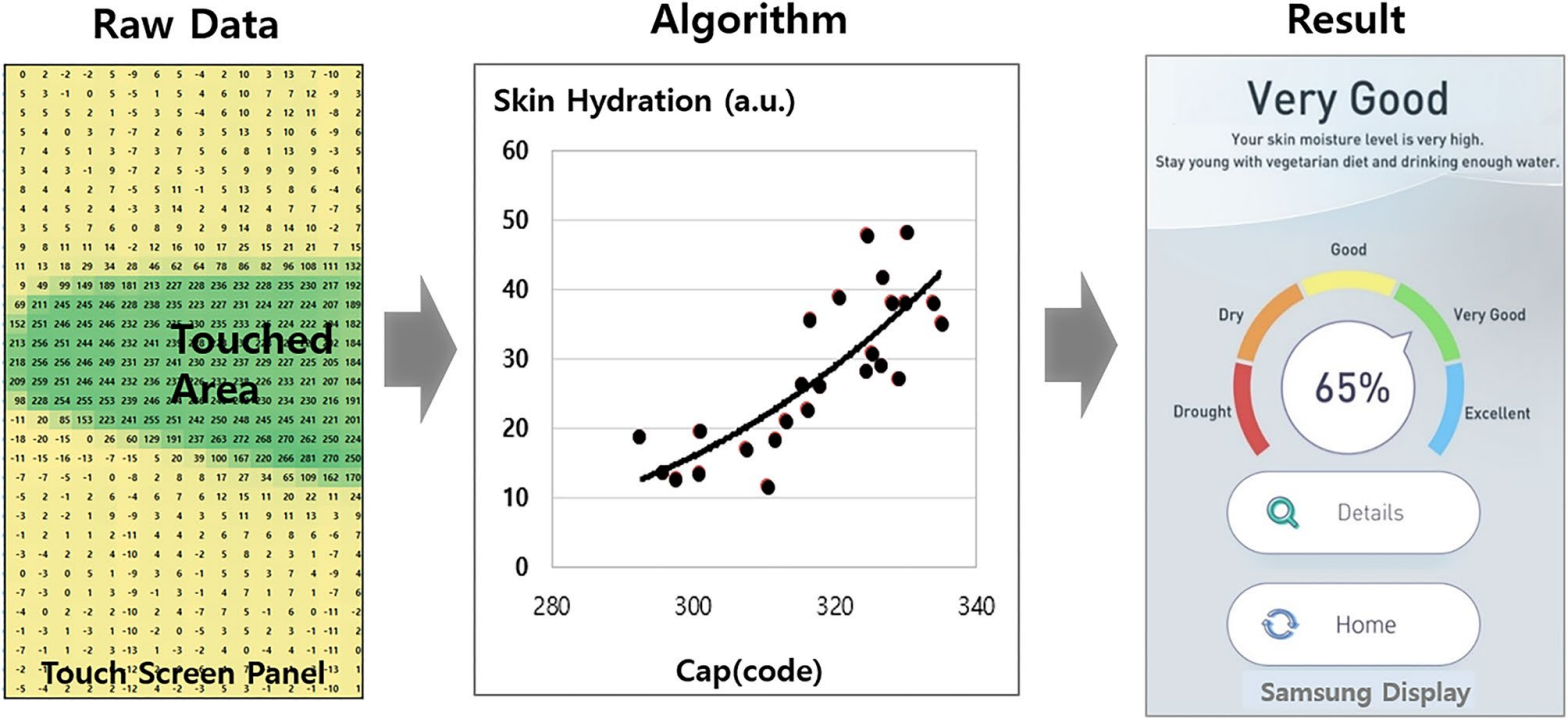
Data processing flow for skin hydration measurement using smartphone.

A smartphone (Galaxy S9, SM-G960N, Samsung Electronics, Suwon, Korea) and Biodisplay (Samsung Display, Yongin, Korea) were used to examine skin hydration (Fig. 2). Changes in capacitance occur in the skin because the human body is mainly composed of water, and the epidermis of the skin is optimized for moisture storage with its specific structure, similar to bricks and mortar^16^. The dielectric constant of water is much higher than that of air, and human skin can highly influence the capacitance variance^17,18^. Based on this principle, the Biodisplay can measure capacitance based on skin moisture. When smartphone users touch their skin onto the touch screen panel, the capacitance is measured at the contact area. The output data were analog to digital converter code values on each pixel, so in this experiment, 528 values in total were presented by a matrix. The Biodisplay algorithm consists of valid capacitance sampling, data mining, and the conversion of capacitance to skin moisture. Valid capacitance data were extracted by applying a threshold in this algorithm, then used to calculate the portion of the touched area. Finally, the capacitance value was applied to a specific equation that converted the capacitance into a measure of skin moisture (Fig. 3).

$$ {\text{Biodisplay}}\,{\text{equation}} = 1.6*\left[ {\left\{ {\left({\text{cap}}\,{\text{.ave}} \right)^{2} /1000} \right\} - 30} \right] $$

** cap.ave: Average of cap code at the contact site.

Biodisplay has been developed with a measurement range of 1 to 100 a.u. and the coefficient of variation (C.V.) of less than ± 10%. The evaluation was conducted to satisfy the specifications when the pressure and temperature change. Biodisplay varied within 3 a.u according to the pressure for all subjects. There was a tendency to vary within the range, which satisfied the C.V. ± 10% specification. When it comes to the influence of temperature on the measurement of the Biodisplay, the maximum of difference was 2 a.u. depending on the temperature. Therefore, the effect on the temperature is very small at a level that satisfied the C.V. ± 10% specification (Supplementary file 1).

#### Hydration probe (HP)

For comparison as a preexisting objective skin hydration measuring device, the HP of DermaLab Combo was used as a gold standard method to measure skin hydration (μS). This HP uses the conductance principle to measure skin hydration. It has a central circular electrode surrounded by eight small pins.

### Procedure

The study was conducted between May 2020 and June 2020. All measurements were performed in a room in which the temperature and the humidity were controlled ((20–22) °C, (45–55) %). Participants were asked to rest for 30 min before the procedure. The skin hydration was measured by two skillful specialists using HP to minimize the influence of investigators like pressure during measurements and the Biodisplay was applied by each participant using their dominant hand. Generally, smartphone users grab their smartphone with their dominant hand and therefore, we measured the skin hydration on the defined point on the arm, nondominant volar arm, 5 cm above the wrist, considering the real setting of measurement using smartphone. All measurements were repeated three times by each device.

First, participants took hold of the smartphone, and made contact between their forearm and the smartphone panel. As the participants maintained contact between their forearm and the smartphone panel, capacitance data on the contact area were recorded for 2 to 3 s, which was the required sensing time. When the measurement was complete, an alarm went off. The skin hydration values were calculated and presented on the mobile display (Fig. 2). All measurements were repeated three times. Then, we measured skin hydration on the forearm three times using HP.

Next, participants applied two fingertip units of moisturizer onto their non-dominant volar forearm, and waited 30 min to allow for absorption. Prior to all measurements, any residual moisturizer should be gently removed through light wiping with a non-woven tissue to ensure the exact evaluation of skin hydration^14^. The Biodisplay is expected to be useful in everyday life, which means people may use it on their skin without washing the measurement area, and they may even use it on their skin over makeup. Therefore, we measured skin hydration both before and after wiping off all residual moisturizer. After 30 min of application, the skin hydration was measured by the Biodisplay and HP three times each in the same manner. Finally, any remaining moisturizer was wiped off from the skin, and the skin hydration was again measured by the Biodisplay and HP three times each.

### Statistical analysis

Statistical analysis was executed using R 4.1.0 (Vienna, Austria; http://www.r-project.org/). The test–retest reliability was assessed using intraclass correlation coefficients (ICC). ICC values were interpreted as (0–0.50), poor agreement; > (0.50–0.75), moderate agreement; > (0.75–0.90), good agreement; and > (0.75–0.90), excellent agreement^19^. A high ICC means that the measurement can be performed reliably by the same person multiple times.

To evaluate the correlation between Biodisplay and HP, Shapiro–Wilk normality test was done first, and Pearson’s correlation analysis (PCC) was performed along with scatter plot^20^. After, PCC was calculated, and *p*-values < 0.05 were considered significant. The range of values for PCC is between (−1 and + 1) (total negative correlation and total positive correlation), where zero values mean no correlation at all. PCC was interpreted as < 0.30: negligible correlation, low positive(negative) correlation (0.30 to 0.50), moderate positive(negative) correlation (0.50 to 0.70), high positive(negative) correlation (0.70 to 0.90) and ≥ 0.90: very high positive (negative) correlation^21^. All statistical analyses were conducted by two biostatistics specialists (SW Kim and JS Shim).

## Supplementary Information

Supplementary Information 1.

Supplementary Information 2.

## Data Availability

The datasets generated during and/or analyzed during the current study are available from the corresponding author on reasonable request.

## Abbreviations

HP: Hydration probe
ICC: Intraclass correlation coefficients
PCC: Pearson’s correlation coefficients
