# Supplementary material for: Novel technology at hand to measure skin hydration by Biodisplay smartphone touch screen panel
Source: Sci Rep. 2021 Sep 30;11:19410. doi: 10.1038/s41598-021-98784-1 (PMC8484361; doi:10.1038/s41598-021-98784-1)
Supplement: Supplementary file 1 — Supplementary Information 1. [file 41598_2021_98784_MOESM1_ESM.pdf]

Novel technology at hand to measure skin hydration

by Biodisplay smartphone touch screen panel

YoungHwan Choi MD, Se Jin Oh MD & Jong Hee Lee MD, PhD

Biodisplay has been developed with a measurement range of 1 to 100 a.u. and the coefficient of variation (C.V.) of less than ±10%. The evaluation was conducted to satisfy the specifications when the pressure and temperature change. The results are as follows.

First of all, on average, the pressure applied to the subject during skin measurement was in the range of 0.5 to 1 kg. Thus, three subjects were repeatedly measured three times each in the corresponding pressure, and the average value of three trials was obtained as shown in Table 1. Biodisplay varied within 3 a.u according to the pressure for all subjects. There was a tendency to vary within the range, which satisfied the C.V. ±10% specification.

[Table 1] The result of average value of cap code and Biodisplay within a range of 0.5 ~1 kg pressure.

Pressure [kg]	Cap.ave [code]		
	Subject 1	Subject 2	Subject 3
0.5	290	285	299
0.7	288	285	301
1	292	288	301
**Pressure [kg]**	**Biodisplay [a.u.]**		
	Subject 1	Subject 2	Subject 3
0.5	87	82	95
0.7	85	82	97
1	88	85	97

Secondly, in order to determine the effect of temperature, the temperature was measured five times using a 3-type calibration checker made of a conductive material. The temperature range was set to 25 ° C and 40 ° C corresponding to the range of use of the skin moisture measuring device used as a golden standard. As shown in Table 2, in case of calibration checker 1, Biodisplay varied from 79(25℃) to 77(40℃), and the maximum of difference was 2 a.u. depending on the temperature. Therefore, the effect on the temperature is very small at a level that satisfied the C.V. ±10% specification.

[Table 2] Biodisplay result dependent on temperature

Calibration Checker	25 ℃	40 ℃
1	79±3	77±3
2	78±2	78±5
3	84±1	83±5
